# Seasonal changes in the hippocampal formation of hoarding and non-hoarding tits

**DOI:** 10.3758/s13420-021-00481-6

**Published:** 2021-08-11

**Authors:** Henrik Lange, Lauren Walker, Markku Orell, Tom V. Smulders

**Affiliations:** 1 Centre for Behaviour and Evolution, Newcastle University, Newcastle upon Tyne, UK; 2 Swedish Environmental Protection Agency, Stockholm, Sweden; 3 Translational and Clinical Research Institute, Newcastle University, Newcastle upon Tyne, UK; 4 Department of Ecology and Genetics, University of Oulu, Oulu, Finland; 5 Biosciences Institute, Newcastle University, Newcastle upon Tyne, UK

**Keywords:** Seasonal changes, Brain plasticity, Memory, Hoarding birds, Caching, Food storing, Paridae, Poecile montanus, Parus major, Hippocampus

## Abstract

The hippocampal formation (HF) processes spatial memories for cache locations in food-hoarding birds. Hoarding is a seasonal behavior, and seasonal changes in the HF have been described in some studies, but not in others. One potential reason is that birds may have been sampled during the seasonal hoarding peak in some studies, but not in others. In this study, we investigate the seasonal changes in hoarding and HF in willow tits (*Poecile montanus*). We compare this to seasonal changes in HF in a closely related non-hoarding bird, the great tit (*Parus major*). Willow tits near Oulu, Finland, show a seasonal hoarding peak in September and both HF volume and neuron number show a similar peak. HF neuronal density also increases in September, but then remains the same throughout winter. Unexpectedly, the great tit HF also changes seasonally, although in a different pattern: the great tit telencephalon increases in volume from July to August and decreases again in November. Great tit HF volume follows suit, but with a delay. Great tit HF neuron number and density also increase from August to September and stay high throughout winter. We hypothesize that seasonal changes in hoarding birds’ HF are driven by food-hoarding experience (e.g., the formation of thousands of memories). The seasonal changes in great tit brains may also be due to experience-dependent plasticity, responding to changes in the social and spatial environment. Large-scale experience-dependent neural plasticity is therefore probably not an adaptation of food-hoarding birds, but a general property of the avian HF and telencephalon.

## Introduction

In food-hoarding Parids (titmice and chickadees), hoarding intensity varies seasonally, typically with a peak in the late summer/autumn and a consistent, but lower intensity period of hoarding throughout winter. Hoarding in these birds is essentially non-existent during the breeding season and summer (Brodin, 1994b; Haftorn, 1954, 1956a, 1956b). A peak in spring is also possible when a sudden abundance of pine seeds occurs (Pravosudov, 1985). The exact timing and duration of the peak(s) in hoarding behavior depend on local environmental conditions, and can therefore vary from location to location (Brodin et al., 1996) and from year to year (reviewed by Pravosudov, 2006).

Seasonal variation in behavior is sometimes accompanied by seasonal changes in the neural substrates associated with these behaviors. This has been well established, for example, in the seasonal changes in the song system in seasonally singing birds (Nottebohm, 1981; Tramontin & Brenowitz, 2000). When studying seasonal changes in particular brain areas, it is important to ascertain that these seasonal changes are specific to the brain area under investigation, and not general changes in the entire brain (or a larger subdivision of the brain). Because almost all these studies are conducted with a between-subject design (individuals need to be killed in order to do histology on the brains), we also need to control for individual variation in brain size within species. This is typically done by also measuring other brain structures and using them as a co-variate in the analysis. Typically used brain areas are the telencephalon, which is the larger brain subdivision that contains most of the song system, as well as the hippocampal formation and many other brain areas, and nucleus Rotundus, a visual processing area in the thalamus. Because these structures may also change seasonally for other (often unknown) reasons (Smulders, 2002), it is important to independently test their seasonal pattern before using them as co-variates to control for individual differences in brain size.

The hippocampal formation (HF) is larger in food-hoarding birds than in non-hoarding birds (Hampton et al., 1995; Krebs et al., 1989; Sherry et al., 1989) and plays an important role in the memory for cache locations (Krushinskaya, 1966; Sherry & Vaccarino, 1989). However, the evidence for seasonal changes in the HF in food-hoarding Parids is less clear (reviewed by Sherry & Hoshooley, 2009, 2010; Sherry & MacDougall-Shackleton, 2015). Smulders and colleagues found seasonal changes in the volume (Smulders et al., 1995) and total cell numbers (Smulders, Shiflett, et al., 2000b) in the hippocampus of black-capped chickadees (*Poecile atricapillus*), with a peak in October, which was believed to be the peak of hoarding behavior in that population of birds near Ithaca, NY, USA (although this was never verified). Barnea and Nottebohm (1994), however, did not find differences in total neuron numbers between wild birds killed in November/December and those killed in March/April near Millbrook, NY, USA (HF volume was not reported). They did, however, find more new neurons being recruited in October/November than in February/March. Hoshooley and colleagues also failed to find seasonal changes in total neurons and HF volume in black-capped chickadees near London, Ontario, Canada (Hoshooley et al., 2007; Hoshooley & Sherry, 2004). These birds had been kept in captivity for 1–2 weeks before the brains were collected. However, in a later study, 1 year later, they found that birds kept in captivity for 6 weeks and killed in April/May had larger hippocampal volumes and total neuron numbers than those killed in November/December (Hoshooley & Sherry, 2007). They also examined the addition of new neurons, and failed to find any seasonal changes in neuronal addition in their captive birds in two of the studies (Hoshooley & Sherry, 2004, 2007), although they found a peak in neuronal recruitment in January in the third study (Hoshooley et al., 2007). Clearly, when it comes to seasonal changes in different attributes of the hippocampal formation in food-hoarding birds, the results are mixed.

Several studies have also investigated seasonal changes in hippocampal volumes in non-hoarding birds. In some nest-parasitic cowbirds, HF is larger at the time of year that females (and sometimes males) are searching for host nests, and only in birds that are involved in searching for host nests (Clayton et al., 1997), but no seasonal changes in volume were detected in brown-headed cowbirds (*Molothrus ater*) (Guigueno et al., 2016). No seasonal changes were found in HF volume in song sparrows (*Melospiza melodia*) (Lee et al., 2001), but house sparrows (*Passer domesticus*) showed a larger HF in the spring than in the autumn, which was the same pattern as shown by black-capped chickadees in that study (Hoshooley & Sherry, 2007). This suggests that seasonal changes in the hippocampus may not be specific to food-hoarding birds. Because all the evidence suggests that hippocampal volume does not respond to typical seasonal signals like photoperiod (Hoshooley et al., 2005; Krebs et al., 1995; MacDougall-Shackleton et al., 2003), the current working hypothesis is that hippocampal volume increases in response to experience. In cowbirds, this may be related to memory use in locating host nests, and in food-hoarding birds, this may be related to the memory formation during hoarding behavior. The variability among studies in food-hoarding birds is then hypothesized to be due to the timing of the sampling of the birds relative to the actual peaks in hoarding intensity in the areas and years in which the birds were sampled, as well as to the birds in some studies being kept in captivity for several weeks, a condition that is known to affect both hippocampal volume (L. B. Day et al., 2008; LaDage et al., 2009; Smulders, Casto, et al., 2000a; Tarr et al., 2009) and neuronal recruitment (Barnea & Nottebohm, 1994; Ladage et al., 2010).

To date, no study of seasonal variation in the hippocampus of hoarding birds has quantified the seasonal changes in hoarding behavior in the same population of birds (Sherry & MacDougall-Shackleton, 2015). Nor has any study looked at seasonal changes in the HF of a closely related non-hoarding species, living in the same area. In the current study, we do both. We quantify the seasonal pattern of hoarding (and eating) intensity in a population of willow tits (*Poecile montanus*) living near Oulu in northern Finland and investigate seasonal changes in the HF of wild willow tits (hoarding) and wild great tits (*Parus major*; non-hoarding), sampled directly from the field. Based on the hypothesis that changes in HF volume are driven by experience, we predict that the HF of willow tits will be larger at the same time of year when we observe a peak in food-hoarding behavior and reduce again in size as hoarding intensity reduces. We do not predict any seasonal changes in the HF of the non-hoarding great tits.

## Methods

### Willow tit behavior

We studied free-living willow tits in Oulu, northern Finland (65°N, 25°30'E). The purpose of collecting hoarding behavior data was primarily to map the autumn peak of hoarding, so that we could plan the collection of brain tissue relative to this hoarding peak the next season. We therefore focused our efforts especially on the period from August to November. Because we had intentions to also collect brains in the spring, mostly to analyze singing and seasonal changes in the song system (Longmoor et al., 2016), we also continued behavioral data collection until birds had started nesting. In the end, we collected data on 79 days between 10 August 2005 and 3 May 2006. We collected behavioral data on average every other day, except for the period from mid-December to late February when no observations were made due to the field conditions being too harsh. The sample sizes for the different months were (in days): 14 (August), 14 (September), 14 (October), 11 (November), 2 (December), N/A (January), N/A (February), 9 (March), 13 (April) and 2 (May). The study population was color-banded, thus mostly allowing individual identification, at least for the winter period. Birds were banded under Finnish Ringing Centre License number 180. This is part of MO's (co-author of this paper) long-term ongoing population study on willow tits. No birds were banded especially for our study. The population study's protocols are to put metal bands on local pulli in the nest during the breeding season. From August to November, a constant banding effort uses mist nets to color band every willow tit they can catch on the field site, thus keeping all birds individually marked. Due to the color-banding effort, the number of individually identifiable birds increased as the season progressed. At banding, age was scored as “adult” or “juvenile” using the shape of the rectrices as criterion (Svensson, 1992). The behavioral data were collected by seeking out bird flocks in their natural habitats in an area of mixed woodland approximately 3 x 2 km in size, without the aid of food supplements or territorial song playback.

Observations took place between 0800 and 1500 h, with the majority before 1300 h. Once located, a flock was followed for as long as possible, usually for about 10–20 min. Behavioral observations focused on a single individual (or occasionally two), following it with the binoculars (10 x 42) for as long as possible. Observations of under 15 s were discarded from the analysis. The mean valid observation duration was 51 s, with a range of 15–420 s. The observations were dictated into a Sony IC recorder and later transcribed into spreadsheets. We strived to sample the behavior of all individuals in a flock (two to six) where possible; however, unmarked individuals were scored as “unknown” and treated as one individual. During an observation we counted the number of occurrences of the different behaviors during that observation bout. An individual's behavior was scored as “foraging” (searching), “eating,” “hoarding,” or “other” (e.g., preening). We recorded single food handling events as either “eating” or “hoarding.”

### Brain collection

Based on the September hoarding peak observed in 2005, we then planned brain collection in such a manner as to minimize the number of birds that needed to be killed and brains to be processed. We decided on collections in July (well before hoarding peak – should be baseline), August (leading up to hoarding peak: might be increasing if HF increases in anticipation to hoarding peak), September (hoarding peak: predict increased HF size), and November (after hoarding peak is definitely over: predict reduced HF size). We also collected brains in the singing season, for analysis of the song system primarily, but we also included these brains in the current analysis. We collected brains from adult (survived at least one winter) willow tits and great tits between July 2006 and September 2007. Birds were captured under a license from the North Osthrobothnian Regional Environmental Centre. Willow tits were caught using mist nets, song playback, and decoy birds in the woods around Oulu from adjacent populations that were not part of the long-term ringed study population. Great tits were caught in the town and suburbs of Oulu, using funnel traps baited with food. A few great tits were caught in the woods (one in September, two in November, and two in March), and a few willow tits were caught in town (two in September and three in April). At capture, birds were kept in cloth bags for a maximum of 2 h 45 min (mean: 1 h 9 min; SD: 31 min) in preparation for processing. Birds were weighed, their wing length and tarsometatarsal length measured, and the amount of fat in the tracheal pit was scored using the ordinal scale recommended by the British Trust for Ornithology (Redfern & Clark, 2001). All birds were aged in the hand based on plumage. Great tits were sexed using the color and pattern of their plumage, and wing length if necessary, and willow tits by the observation of song production and wing length. Sex was confirmed after the dissection of the gonads. The sample sizes were as follows: July 2006/2007 (WT: 3 F/5 M, GT: 4 F/4 M), August 2006/2007 (WT: 4 F/3 M, GT: 4 F/5 M), September 2006/2007 (WT: 4 F/6 M, GT: 4 F/4 M), November 2006 (WT: 3 F/4 M, GT: 2 F/6 M), and spring 2007. In the spring breeding season, great tits were collected between 24 March and 17 April 2007 (4 F/4 M), and willow tits were collected between 16 April and 22 April 2007 (2 F/6 M). The average timing of the first clutches in 2007 was 15 May for great tits, and 10 May for willow tits.

### Tissue preparation

Birds were processed in the field in the back of a Land Rover®, especially equipped for this purpose. They were humanely killed by rapid decapitation. One hemisphere of the brain was immersed in 4% paraformaldehyde in PBS, while the other hemisphere was fresh frozen on dry ice. Which hemisphere was fixed and which was fresh frozen was alternated among birds, so half the birds for each time point, species, and sex had the right hemisphere fixed, and half the left hemisphere. We only report on the fixed hemisphere in this study. After 48 h of fixation, the hemispheres were cryoprotected in 30% sucrose solution, embedded in O.C.T. (Optimal Cutting Temperature compound for cryosectioning), frozen on dry ice, and stored at −80 °C. After all of the samples had been collected, they were shipped from Oulu to Newcastle. Coronal sections of 70 μm were cut on a cryostat (Microm HM560), and every other section was thaw-mounted onto gelatin-coated slides. The sections were stained with cresyl violet and coverslipped with Histomount®.

### Microscopy and quantification

The person quantifying the slides (HL) was blind to the identity of the birds he was working on (although the species is easily determined from just looking at the sections). Slides were viewed using a Leica DM-LB microscope with a motorized stage, and connected to a computer running StereoInvestigator v7.5 (MBF Bioscience, Williston, VT, USA). The telencephalon was outlined at 2.5X magnification on every eighth section (560 μm apart). Nucleus rotundus was outlined every fourth section (280 μm apart). Finally, the HF was outlined every fourth section (280 μm apart), and cells counted using the Optical Fractionator in StereoInvestigator at 100X (oil immersion) magnification. The counting parameters were as follows: counting frame was 30 x 30 μm, sampling grid was 450 x 450 μm, and dissector height was 25 μm. To calculate volumes, measured areas were multiplied by the distance between measured sections and added up. We counted two cell types in the HF, identified as follows: neurons are defined as large cells with a clear (low-staining) nucleus and obvious, darkly stained nucleoli; small cells are smaller than neurons and have a darkly stained nucleus with or without obvious nucleoli. The small cells could be neurons or non-neuronal cells, like glia.

### Data analysis

For the behavioral data, sometimes the same individual was observed for more than one observation in a given day. For the analysis, we combined all the observations of any given individual on a particular day and calculated the rate of eating and hoarding per hour of observation for that individual by dividing its total number of hoarding or eating events by its total observation time for that day. The per-hour rates for all individuals observed in a given day (on average about nine individuals: three individuals in each of three flocks) were then averaged to give a daily hoarding and eating rate. These rates per hour were rounded to the nearest whole number to give a number of items per hour. Statistical analyses were performed with these daily rates as the unit of analysis using Generalized linear models (GzLM) in IBM SPSS 26®. We tested for fit to a Poisson distribution, and for both variables (eating and hoarding), this was severely overdispersed. We therefore fit a Negative Binomial Distribution with log link function, allowing SPSS to estimate the Negative Binomial parameter from the data. This gave a better fit (AIC was lower) than the default parameter of 1. Fat reserves, because they are scored on an ordinal scale, were treated as multinomial ordinal data with cumulative logit link function in the GzLM. To analyze the neurobiological variables, we also used GzLM, using linear response variables. All anatomical variables were natural-log transformed to better reflect the log-log allometric relationships between parts of the body and brain. Analysis of brain structures is always controlled for individual variation in either body size (tarsometatarsus length) or in telencephalon size in the analysis of Rt, HF volume, and cell numbers, by using that variable as a linear co-variate in the analysis. No interactions with the co-variate are included in the model. For the cell type analyses, we used Generalized Estimating Equations, with cell type as a within-subject factor. Otherwise, this was treated exactly as the other neurobiological variable analyses. The test statistic for these models is Wald’s Χ^2^. Pair-wise post-hoc comparisons were performed using the Least Significant Difference method. Findings are considered significant if p < 0.05.

## Results

### Willow tit behavior

**Fig. 1:**
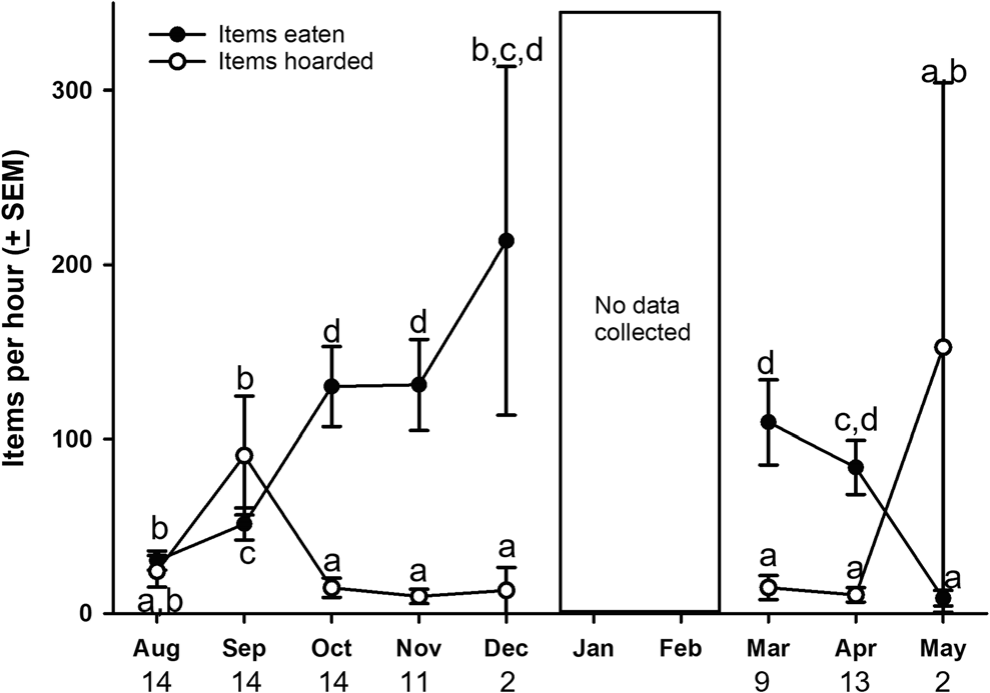
Seasonal variation in eating rate (filled symbols) and hoarding rate (open symbols) of willow tits. All rates are expressed as items per hour and represent means (+ SEM) across all the observation days for that month. Different letters indicate months that are significantly different from each other in pairwise comparisons. Letters should only be compared within a behavior, not across behaviors. Numbers under the X-axis represent the sample size for that month (number of days)

The willow tits' rate of eating (expressed as events per hour) varied over the year with the highest rates from October to April and the lowest in May (Χ^2^_7_ = 72.11, p < 0.0005). Hoarding intensities varied across the year and we found a clear hoarding peak in September (Χ^2^_7_ = 27.29, p < 0.0005). Pairwise comparisons are indicated in Fig. 1.

### Body size

The length of the tarsometatarsal bone is used as an index of body size, as it is not plastic in adulthood. Great tits have longer tarsometatarsals (Χ^2^_1_ = 1,206.32, p < 0.0005) than willow tits and males of both species have longer tarsometatarsals (Χ^2^_1_ = 28.60, p < 0.0005) than females. The interaction between sex and species was just not significant (Χ^2^_1_ = 3.62, p = 0.057). There are no significant differences across the different times of year for tarsometatarsus length (Χ^2^_4_ = 5.20, p = 0.27), nor are there any significant interactions with season (all p > 0.19).

**Fig. 2:**
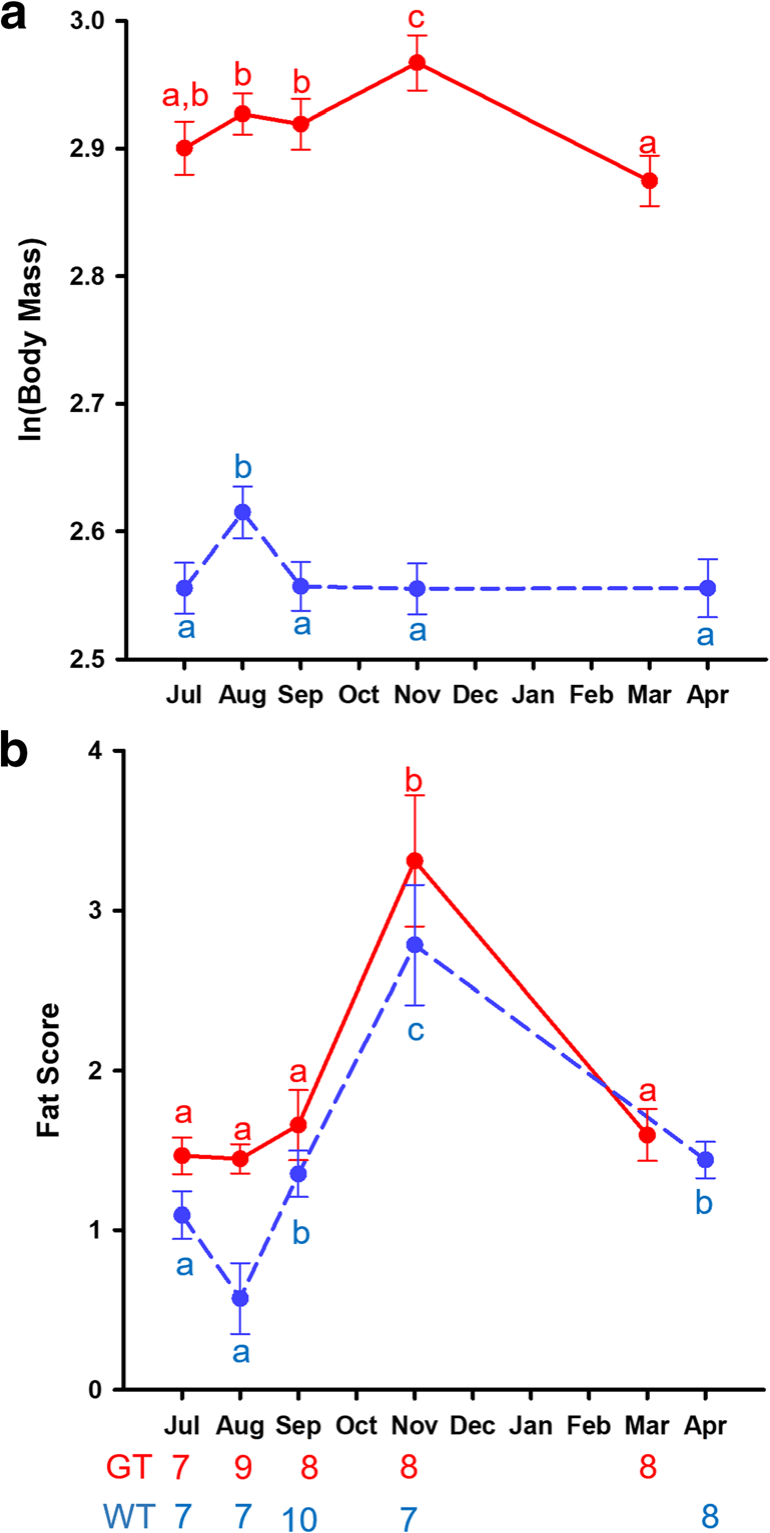
Seasonal patterns in (**a**) body mass and (**b**) fat scores. Body mass is controlled for tarsometatarsal length. The ln(body mass) values plotted are the estimated marginal means with ln(tarsometatarsal length) kept constant at 2.95. The fat score values are means of the ordinally scored variable. Red (solid line): great tits, blue (stippled line): willow tits. Error bars represent standard errors of the mean, and letters represent pairwise comparisons within a species. Different letters indicate significant differences. The numbers under the bottom X-axis represent the sample sizes for each month and species

Tarsometatarsal length significantly predicted body mass (Χ^2^_1_ = 18.43, p < 0.0005). When controlling for tarsometatarsal length, relative body mass was significantly higher for great tits than for willow tits (Χ^2^_1_ = 119.82, p < 0.0005), and significantly higher in males than in females (Χ^2^_1_ = 33.69, p < 0.0005), without an interaction between the two factors (Χ^2^_1_ = 0.15, p = 0.70). There was a significant effect of season (Χ^2^_4_ = 25.71, p < 0.0005), as well as a significant interaction between species and season (Χ^2^_4_ = 18.29, p = 0.001). Great tits were significantly heavier in November than any other times of the year, and significantly lighter in March than in August. Willow tits, in contrast, were significantly heavier in August than at any other time of the year (Fig. 2a). There were no other significant interactions (all p > 0.29).

Great tits generally carried more fat reserves than willow tits (Χ^2^_1_ = 12.35, p < 0.0005) and birds of both species carried more fat in November than at any other time point (Χ^2^_4_ = 39.62, p < 0.0005). There was no significant interaction between species and season (Χ^2^_4_ = 39.62, p < 0.0005), but the pairwise comparisons indicated in Fig. 2b are from separate analyses for each species. There was no significant effect of sex, nor any significant interactions with sex.

### Telencephalon volume

**Fig. 3:**
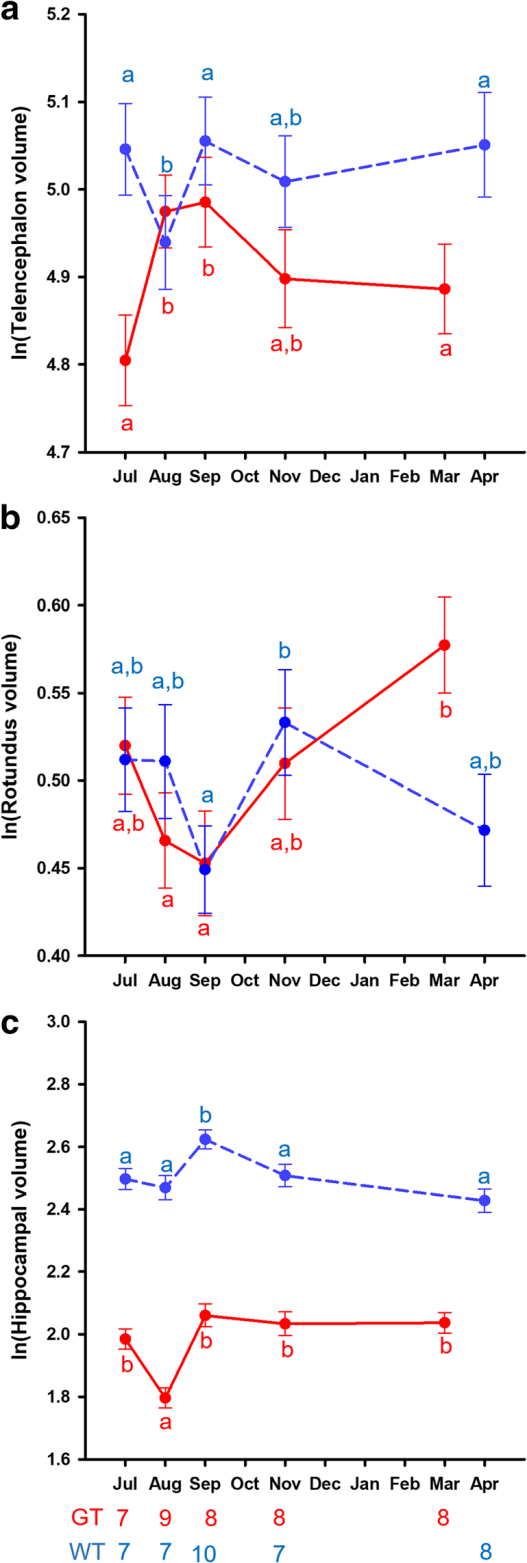
Seasonal patterns in (**a**) telencephalon volume, (**b**) nucleus rotundus volume, and (**c**) hippocampal formation (HF) volume. Telencephalon volume is controlled for tarsometatarsal length, and rotundus and HF volumes are controlled for telencephalon volume. The ln(telencephalon volume) values plotted are the estimated marginal means with ln(tarsometatarsal length) kept constant at 2.96. The ln(rotundus volume) values are estimated marginal means, with ln(telencephalon) kept constant at 4.97. The ln(hippocampal volume) values are estimated marginal means with ln(telencephalon) kept constant at 4.97. Red (solid line): great tits, blue (stippled line): willow tits. Error bars represent standard errors of the mean, and letters represent pairwise comparisons within a species. Different letters indicate significant differences. The numbers under the bottom X-axis represent the sample sizes for each month and species

Tarsometatarsal length significantly predicts Tel volume (Χ^2^_1_ = 8.16, p = 0.004). Both species and sexes have similar telencephalon size relative to body size (species: Χ^2^_1_ = 1.76, p = 0.18; sex: Χ^2^_1_ = 0.46, p = 0.50). There is a small main effect of time of year (Χ^2^_4_ = 10.00, p = 0.040), but there is a strong significant interaction between species and time of year (Χ^2^_4_ = 19.72, p = 0.001). Willow tit telencephalon is significantly smaller in August than at any other time of year, except November, while great tit telencephalon significantly increases from July to August and September, and then reduces again in size to November and March (Fig. 3a). No other interactions were significant (all p > 0.11).

### Nucleus rotundus volume

In one willow tit from the July sample, nucleus rotundus (Rt) was too damaged to get a reliable estimate of its size. All analyses that include Rt therefore exclude this bird. Telencephalon significantly predicts Rt size (Χ^2^_1_ = 14.77, p < 0.0005), and there are no species (Χ^2^_1_ = 0.22, p = 0.64) or sex (Χ^2^_1_ = 2.06, p = 0.15) differences in relative Rt size, but there is a small seasonal effect (Χ^2^_4_ = 9.69, p = 0.046), with September birds of both species having a slightly smaller Rt size compared to other times of the year (Fig. 3b).

### Hippocampal volume

Telencephalon size significantly predicts HF volume (Χ^2^_1_ = 41.66, p < 0.0005), and willow tits have a larger relative HF than great tits (Χ^2^_1_ = 436.64, p < 0.0005), but there are no sex differences (Χ^2^_1_ = 0.14, p = 0.71), nor a significant interaction between the two factors (Χ^2^_1_ = 3.57, p = 0.059). There are significant seasonal changes (Χ^2^_4_ = 41.09, p < 0.0005), the patterns of which differ between the two species (interaction: Χ^2^_4_ = 17.89, p = 0.001; Fig. 3c). In great tits, the relative hippocampus size is smaller in August than at any other time of the year (p < 0.0005). Given the significant increase in telencephalon size in great tits at this time, this means that HF volume follows telencephalon volume, but with a lag, so that HF volume is still smaller in August when telencephalon volume has already increased. There is also a three-way interaction between species, sex and time of year (Χ^2^_1_ = 14.36, p = 0.006). Pair-wise comparisons indicate that in male great tits (but not in female great tits, nor in willow tits) there is a peak in relative HF volume in November, which is significantly larger than July and March (both p < 0.032). This may again indicate a delay in following the changes in telencephalon volume, at least in males. In all willow tits, relative HF volume is significantly larger in September than at any other time of the year (all p < 0.012), while male willow tits have a secondary, smaller peak in July that females do not have. This male peak is significantly higher than August and April (both p < 0.007).

### Hippocampal cell numbers

**Fig. 4:**
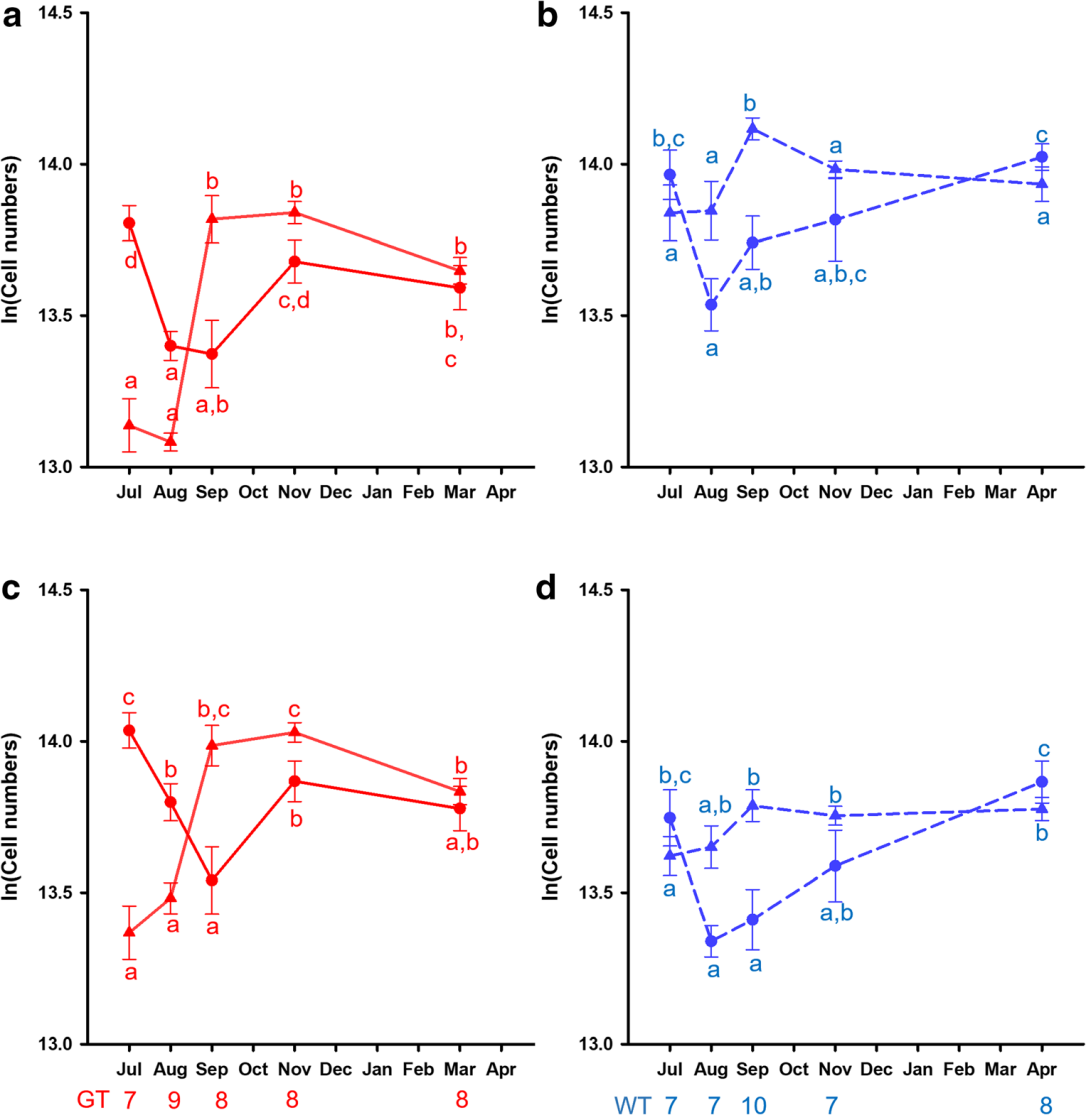
Seasonal patterns in (**a**, **c**) great tit and (**b**, **c**) willow tit cell numbers in the hippocampal formation (HF). Numbers in A and B are controlled for telencephalon volume, while those in C and D are controlled for HF volume (effectively making these numbers equivalent to densities). The values plotted are estimated marginal means, with ln(telencephalon) kept constant at 4.97 (**a, b**) and ln(HF) kept constant at 2.25 (**c, d**). Red (solid line): great tits, blue (stippled line): willow tits; triangles: neurons, circles: small cells. Error bars represent standard errors of the mean, and letters represent pairwise comparisons within a species. Different letters indicate significant differences. The numbers under the bottom X-axis represent the sample sizes for each month and species

Willow tits have on average 2.16 + 0.06 *10^6^ cells in their HF, compared to 1.65 + 0.06 *10^6^ cells for great tits. The mean Coefficients of Error (Gundersen, m = 1) on these estimates are 2.3 + 0.07% for willow tits and 2.7 + 0.08% for great tits. We conducted a Generalized Estimating Equations analysis, with cell type as the within-subject variable, and (ln-transformed) Telencephalon volume as a co-variate. Telencephalon volume significantly predicted total HF cell numbers (Χ^2^_1_ = 15.02, p < 0.0005). Across both species, females have marginally more cells in the HF than males (Χ^2^_1_ = 4.00, p = 0.045). Willow tits have more cells in the HF than great tits (Χ^2^_1_ = 65.96, p < 0.0005), and there was no significant difference in the numbers of the two cell categories (Χ^2^_1_ = 1.091, p = 0.30). There were significant seasonal differences (Χ^2^_4_ = 52.13, p < 0.0005), as well as interactions between species and cell type (Χ^2^_1_ = 10.02, p = 0.002), season and cell type (Χ^2^_4_ = 60.92, p < 0.0005), and species, season, and cell type (Χ^2^_4_ = 41.77, p < 0.0005; Fig. 4a and b). There is also a significant three-way interaction between species, sex, and cell type (Χ^2^_1_ = 6.64, p = 0.01). No other interactions are significant (all p > 0.076).

To understand how the changes in cell numbers relate to the changes in volume, we then use HF volume as a co-variate instead of telencephalon volume. This in effect tells us about changes in densities of the different cell types. As expected, HF volume significantly predicted cell numbers (Χ^2^_1_ = 54.77, p < 0.0005). Unlike cell numbers, great tits have a higher density of cells than willow tits (Χ^2^_1_ = 4.51, p = 0.034), and this differs significantly by cell type (interaction: Χ^2^_1_ = 10.02, p = 0.002), with willow tits (but not great tits) having a lower density of small cells than neurons. There are significant changes in cell density across seasons (Χ^2^_4_ = 55.80, p < 0.0005) and this differs depending on species (interaction: Χ^2^_4_ = 19.07, p = 0.001), cell type (interaction: Χ^2^_4_ = 60.92, p < 0.0005), and both (three-way interaction: Χ^2^_4_ = 41.77, p < 0.0005; Fig. 4c and d). Females have a slightly higher cell density than males overall (Χ^2^_1_ = 4.97, p = 0.026), but this only applies to neurons in great tits, and to small cells in willow tits (three-way interaction between species, sex, and cell type; Χ^2^_1_ = 6.64, p = 0.01).

To understand the three-way interactions between season, species, and cell type for both cell numbers and cell densities, we looked at the seasonal pairwise comparisons in each species and cell type (indicated as letters in Fig. 4). Great tits have more neurons in September, November, and March than in July and August, and slightly fewer in March than in November (p < 0.0005) when accounting for telencephalon volume, and this pattern is the same when accounting for hippocampal volume. This means that neuron number and density changes strongly in great tits, remaining high even after HF volume reduced again, following changes in telencephalon volume. In willow tits, neuron numbers are larger in September than at any other time of the year (pairwise comparisons: all p < 0.009). When we account for hippocampal volume, however, neuron densities are lowest in July and increase towards September. They do not decrease again in November, however, but stay the same through April.

The numbers of small cells showed a very different pattern from the neurons and this pattern is similar in both species. The number of small cells drops significantly from July to August in both great and willow tits (p < 0.0005), and then gradually increases back in the spring. In willow tits, the density of small cells follows exactly the same pattern as the numbers. However, in great tits the density first gets smaller in September, when the volume increases dramatically, but the numbers do not. After that, densities gradually increase to the spring, same as the numbers.

## Discussion

This study, for the first time, quantified both the seasonal changes in hoarding intensity and seasonal changes in hippocampal formation in the same population of a hoarding bird: the willow tit. We found that hippocampal volume is indeed larger at the time of year that hoarding intensity is highest, and only at that time of the year. This increased volume is accompanied by an increase in neuron numbers and density in the HF. Neuron density remains high throughout winter, even after volume and total number decreases again. Surprisingly, we also found seasonal changes in the HF of a closely related non-hoarding bird, the great tit. The pattern of seasonal changes was different from that of the willow tits. Unlike willow tits, great tits showed significant seasonal changes in telencephalon volume, with a peak in August and September. Great tit HF volume followed this change in telencephalon volume, although with a slight delay, resulting in a relatively smaller HF in August. The increase in HF volume from August to September was reflected in an increase in both number and density of neurons. Although HF volume follows the Tel volume decrease in November, neuron density and numbers stay higher all through winter than they were in summer. In both species, the small cells followed a completely different pattern, with fewer of them throughout winter, and more in spring and summer.

### Seasonal changes in behavior

We did not quantify eating behavior in great tits. Although we did not collect data in the very coldest months, willow tits were shown to eat most intensely when days are shorter and colder, which means there is less time to eat the food needed to survive the longer, colder nights. This is also the time of year that both species carried the most fat reserves (November sample), and that great tits were the heaviest. Willow tit relative body mass stayed the same throughout winter, bar a small increase in August, when fat reserves were the lowest. We do not know why this might be the case, especially since fat reserves are higher in winter, and others have shown that closely related species were also heavier in winter, with larger muscle mass, as well as other organs (Liknes & Swanson, 2011; Petit et al., 2014). At best, we can speculate that our November willow tits might have had empty digestive tracts, hiding the increase in body mass due to increased fat reserves.

Hoarding intensity is highest in September, and possibly also in May (although this was not significant, since we only had 2 days of sampling in May). This seasonal pattern in hoarding behavior is consistent with other observations in the literature (Pravosudov, 2006). We hypothesize that the seasonal pattern of hoarding intensity observed in the field is the result of a seasonal pattern in food availability: high in September (and sometimes in the Spring; Pravosudov, 2006), but lower during the rest of winter, combined with high hoarding motivation from the end of summer to the end of winter. What then drives hoarding motivation? Our data do not give any new insights into motivation to hoard, but the literature suggests that hoarding motivation does not seem to be regulated by photoperiod in the same way that reproductive behavior is, although shorter days do seem to be associated with, and to induce, hoarding under certain conditions (Krebs et al., 1995; MacDougall-Shackleton et al., 2003; Shettleworth et al., 1995). This might be due to the reduced access time to food as days are shorter (and therefore the increased need to use fat reserves to get through the longer night). A direct test of this hypothesis showed that whereas fat reserves responded as expected to shorter days (more fat reserves for longer nights), hoarding behavior did not respond to this manipulation in captivity (Karpouzos et al., 2005). However, in that study, the temperature was at 19 °C throughout day and night. Bartness and colleagues have proposed for Siberian hamsters that hoarding motivation may respond to energy balance and the use of fat reserves (“energy flux”) (D. E. Day & Bartness, 2001; Keen-Rhinehart et al., 2010). Motivation would then increase as nights get longer and (importantly) colder (causing more use of fat reserves) and decrease again when nights get shorter and warmer. This hypothesis remains to be tested in Parids.

### Seasonal changes in the willow tit HF

We find a peak in HF volume in willow tits that coincides with the peak in hoarding intensity in the field (in September). HF volume is significantly smaller, with fewer neurons, only a month earlier (in August) and again 2 months later (in November). If we assume that it takes several weeks to increase to size of the HF, this finding supports the hypothesis that HF volume changes in response to hoarding intensity, rather than in anticipation of hoarding intensity, and that it decreases when hoarding intensity decreases. Our working hypothesis is that hoarding birds form a memory of every item they hoard. This memory is used to avoid caching new items too close to existing caches (Male & Smulders, 2007b), as well as for retrieval of these items within a few days to weeks from hoarding them. All the evidence suggests that the memory for individual cache locations does not last throughout winter (Brodin, 1994a, 2005; Hitchcock & Sherry, 1990; Male & Smulders, 2007a), and theoretical models have shown that food can be beneficial throughout winter without explicit memory for cache locations (Smulders, 1998). We therefore hypothesize that the formation of thousands of new spatial memories over a short period of time drives a temporary increase in hippocampal volume, and the decay of these memories is accompanied by a shrinking of the HF.

This hypothesis can also explain the inconsistencies between previous studies on seasonal changes in HF volume in food-hoarding birds: if these birds were sampled away from the hoarding peak (e.g., Barnea & Nottebohm, 1994, injected their birds in October, but sampled them in November/December), no change in HF volume or cell numbers would be observed. And all the studies conducted around London, ON, Canada kept the birds in captivity for at least a few weeks (Hoshooley et al., 2007; Hoshooley & Sherry, 2004, 2007), which may have reduced their hippocampal volumes significantly (LaDage et al., 2009; Smulders, Casto, et al., 2000a; Tarr et al., 2009). The ultimate test of this working hypothesis would be to either sample birds during a spring peak in hoarding intensity or to induce high hoarding intensity in early spring by food supplementation. We would predict that, as long as the intensity was kept up for long enough (4–5 weeks), we should see an increase in HF volume.

The volume changes were accompanied by increases in the numbers of neurons. This is similar to what we found in a previous study in black-capped chickadees, when we did not use stereological techniques (Smulders, Shiflett, et al., 2000b). Similar to that study, we found that the density of neurons increased from summer to the autumn, as the volume increases as well. However, as the volume decreases again, the neuron numbers go down, but the density of neurons remains unchanged throughout winter. One way this pattern could be explained is by growth of existing neurons. Indeed, we find that our smallest cell category, presumably made up of smaller neurons and glia, show lower numbers and densities in the autumn than in the spring and summer. This might imply that some small cells may become larger cells come autumn, and that the reversion (shrinkage) of these cells happens slowly as the number of larger neurons decreases with decreasing volume from September to November. However, given the findings of Barnea and Nottebohm (1994) showing increased neuronal recruitment in the chickadee HF in the autumn, neuronal addition may also play a role in these volumetric changes and changes in neuron numbers.

### Seasonal changes in the great tit brain

Perhaps the largest surprise to us was the strong seasonal variation in the great tit brains. Telencephalon volume increased in August and September and decreased a bit after that to November and April. HF volume seemed to follow this same pattern, but with a bit of a lag: HF is smaller than expected from telencephalon size in August, but larger than expected from telencephalon size in November (in males only). The neuron numbers and densities show an even stronger pattern, being lower in the summer (July and August) than in autumn and spring (September, November, April). Like in the willow tits, numbers and density of small cells decrease around the time that numbers of larger neurons increase, suggesting that cell growth may account for at least some of this pattern.

We did not quantify any great tit behavior, but we know that great tits’ social structure changes in the non-breeding season. At the end of the summer, great tits stop being territorial and start using larger home ranges, which they roam in larger fission-fusion flocks (Ekman, 1989; Matthysen, 1990). This change in spatial and social environment may require the animals to process a lot of new information at the end of the summer, including the different locations of food sources in a much larger home range (possibly responsible for seasonal changes in HF), and their own standing in the more complex social group (possibly responsible for seasonal changes in the rest of the telencephalon). Whatever the explanation for the great tit seasonal pattern in telencephalon size and hippocampal neuron numbers, it is different from that in the willow tit brains, lending more support to the idea that the changes in the willow tit HF are indeed related to the hoarding behavior, and are not a more general response to the changes in weather and food availability experienced by all species in that environment.

### Neural plasticity in birds

The fact that we find changes in both species, some in entire telencephalon volume, others specific to HF, is consistent with the idea that the avian brain in general may be especially plastic in its responses to environmental challenges. We found that Rt was smallest in September, which is consistent with a meta-analysis showing Rt to be smaller in the non-breeding season in songbirds (Smulders, 2002), although no functional reason for this has been put forward yet. Changes in the size of the telencephalon, like those we report here for great tits, have also been reported in some studies, although the direction seems to depend on the species and/or the conditions (Smulders, 2002).

The seasonal changes in the number of HF neurons in both species, associated with changes in hoarding behavior for willow tits, and potential changes in space use for great tits, also suggests that adult hippocampal plasticity is not a specific adaptation of food-hoarding birds, but is a more general phenomenon. It is true that during development, the HF of food-hoarding birds is more sensitive to spatial experience than that of non-hoarding birds (Clayton, 1995). However, this same (small) amount of experience that seems to trigger big changes in hippocampal volume during development is not able to change hippocampal volume in adulthood (Cristol, 1996; Krebs et al., 1995; MacDougall-Shackleton et al., 2003). In fact, no captive experience has yet been found to increase hippocampal volume, although it has been shown to affect hippocampal neurogenesis (LaDage et al., 2009, 2010). Other cases of (seasonal) hippocampal plasticity in the wild can probably also be explained by an experience-dependent mechanism driving hippocampal neuron sizes and/or numbers, and therefore changes in hippocampal volume (Clayton et al., 1997; Healy et al., 1996; Pravosudov et al., 2006). The fact that this cannot be replicated in the lab is either because this amount of experience cannot be replicated in captivity, or because the stress of captivity itself counters the effects of experience (LaDage et al., 2009; Smulders, Casto, et al., 2000a; Tarr et al., 2009).

## Conclusion

In this study, we confirm that the hippocampus of food-hoarding Parids (willow tits) changes seasonally in its volume and number of (large) neurons, and that this seasonal change is closely linked to the seasonal changes in food-hoarding intensity. This is consistent with the hypothesis that the increase in HF volume is driven by the intense spatial memory formation associated with high-intensity hoarding. In addition, we show that non-hoarding great tits’ brains also change seasonally, although in a different pattern to that of the willow tits. The increase in telencephalon volume (including a concomitant, though delayed, increase in HF volume) and the strong increase in the number of large HF neurons is correlated with a presumed increase in social network and home range size. This is consistent with the results of food-hoarding birds, suggesting that intense use of the hippocampus can increase its large neuron numbers. This suggests that seasonal plasticity in the HF (and indeed in other telencephalic areas) may not be a species-specific adaptation of hoarding birds and brood parasites, but a more general feature of the avian hippocampal formation, or even the entire telencephalon.
